# Entropy and Normalization in MCDA: A Data-Driven Perspective on Ranking Stability

**DOI:** 10.3390/e28010114

**Published:** 2026-01-18

**Authors:** Ewa Roszkowska

**Affiliations:** Faculty of Computer Science, Bialystok University of Technology, Wiejska 45A, 15-351 Bialystok, Poland; e.roszkowska@pb.edu.pl

**Keywords:** Multiple-Criteria Decision Analysis, normalization techniques, TOPSIS, entropy, ranking stability, rank reversal, data distribution, cost and benefit criteria, uncertainty

## Abstract

Normalization is a critical step in Multiple-Criteria Decision Analysis (MCDA) because it transforms heterogeneous criterion values into comparable information. This study examines normalization techniques through the lens of entropy, highlighting how criterion data structure shapes normalization behavior and ranking stability within TOPSIS (Technique for Order Preference by Similarity to Ideal Solution). Seven widely used normalization procedures are analyzed regarding mathematical properties, sensitivity to extreme values, treatment of benefit and cost criteria, and rank reversal. Normalization is treated as a source of uncertainty in MCDA outcomes, as different schemes can produce divergent rankings under identical decision settings. Shannon entropy is employed as a descriptive measure of information dispersion and structural uncertainty, capturing the heterogeneity and discriminatory potential of criteria rather than serving as a weighting mechanism. An illustrative experiment with ten alternatives and four criteria (two high-entropy, two low-entropy) demonstrates how entropy mediates normalization effects. Seven normalization schemes are examined, including vector, max, linear Sum, and max–min procedures. For vector, max, and linear sum, cost-type criteria are treated using either linear inversion or reciprocal transformation, whereas max–min is implemented as a single method. This design separates the choice of normalization form from the choice of cost-criteria transformation, allowing a cleaner identification of their respective contributions to ranking variability. The analysis shows that normalization choice alone can cause substantial differences in preference values and rankings. High-entropy criteria tend to yield stable rankings, whereas low-entropy criteria amplify sensitivity, especially with extreme or cost-type data. These findings position entropy as a key mediator linking data structure with normalization-induced ranking variability and highlight the need to consider entropy explicitly when selecting normalization procedures. Finally, a practical entropy-based method for choosing normalization techniques is introduced to enhance methodological transparency and ranking robustness in MCDA.

## 1. Introduction

Multiple-Criteria Decision Analysis (MCDA) is a fundamental methodological framework for evaluating and ranking alternatives characterized by multiple, often conflicting criteria [1,2]. MCDA has been widely applied in the social sciences, including economics, management, business, finance, and public policy [3,4,5,6,7], among others.

The MCDA process typically includes constructing a decision matrix, normalizing criteria, weighting, and aggregating into a final preference score [1,8]. Among these steps, normalization is pivotal. It allows for a comparison of criteria measured on different scales and directly affects both the stability and interpretability of ranking results [9,10]. Normalization is not a trivial methodological choice but a decision-analytic component with substantive implications. Normalization interacts with the intrinsic characteristics of the data. This interaction can further influence ranking outcomes. Criteria with larger magnitudes or broader numerical ranges may disproportionately affect aggregation results if normalization is inadequately selected. This can lead to biased rankings and potentially misleading decisions [11,12,13,14].

Consequently, normalization is increasingly viewed not merely as a technical preprocessing step, but as a decision-analytic component with substantive implications for ranking outcomes, robustness, and rank reversal phenomena [15,16,17,18]. In this sense, normalization contributes to decision-making uncertainty, since alternative normalization procedures may generate different rankings even when all other elements of the MCDA model remain unchanged. This highlights uncertainty as an inherent methodological feature of MCDA rather than simply stemming from data constraints.

Over the past two decades, a substantial body of research has examined normalization techniques in MCDA, addressing their formal properties [11,19], effects on rankings [20,21,22], and robustness across decision contexts [15,23,24,25]. Despite its recognized importance, the choice of normalization technique in many MCDA applications is rarely theoretically justified, and sensitivity analyses with respect to normalization are often omitted or only superficially discussed.

Entropy, introduced by Shannon [26], quantifies uncertainty or dispersion within a dataset. In this study, uncertainty is understood in a structural sense, arising from the informational heterogeneity of criterion data and its interaction with normalization procedures. In MCDA, entropy is often employed as an objective weighting technique. Criteria with higher variability and lower entropy are assigned higher weights. Criteria with more uniform values receive lower weights [27,28]. Extensions such as Tsallis [29] and Rényi entropy [30] have been proposed to handle incomplete, noisy, or highly complex decision data. Beyond weighting, entropy provides insight into the structural properties of criterion data. It reflects the intrinsic informational content of a criterion and distinguishes between low-entropy criteria, which exhibit pronounced disparities and high discriminatory potential, and high-entropy criteria, which show near-uniform values and limited discrimination power. This distinction is particularly important for normalization, as different techniques respond differently to data dispersion, extreme values, and skewness.

Despite extensive research, a methodological gap remains: the role of entropy as a mediator between data structure and normalization behavior has not been systematically examined. Specifically, it is unclear how criteria with different entropy levels respond to various normalization procedures and how this interaction affects ranking robustness.

This study addresses this gap by providing a structured theoretical and empirical analysis of normalization techniques in MCDA, explicitly considering the influence of criterion entropy on normalization outcomes. Rather than using entropy as a weighting mechanism, we treat it as a descriptive measure of data distribution, which helps explain and interpret the behavior of normalized values and resulting rankings.

This study addresses the following research question:

*How does the entropy level of criterion data influence the behavior of different normalization techniques and the resulting ranking stability in MCDA methods such as TOPSIS?*

Normalization techniques are not distribution-neutral transformations. Their effects depend critically on the underlying data structure, particularly on the degree of dispersion and the presence of extreme values. Ranking stability is understood here as the consistency of alternative ordering under different normalization techniques, measured through rank correlation and rank reversal analysis.

To illustrate the proposed framework, an empirical experiment is conducted using a decision matrix of ten alternatives and four criteria, comprising two high-entropy and two low-entropy criteria. Seven widely used normalization procedures are applied, including vector normalization, max normalization, linear sum normalization, linear cost inversion, reciprocal cost transformation, and linear max–min normalization. Cost-type criteria are handled according to the specific formulation of each normalization method. This controlled example allows for examining how entropy and cost treatment interact with normalization, providing a practical context for the theoretical analysis without implying that the observed effects are universally generalizable.

This study contributes to the literature in several ways. First, it synthesizes existing normalization research by organizing methods according to their mathematical properties and sensitivity to data distribution. Second, it introduces entropy as a unifying descriptive concept linking raw data structure with normalization behavior and ranking stability. Third, it provides a systematic comparative analysis of selected normalization techniques and illustrates their effects through an empirical example based on TOPSIS (Technique for Order Preference by Similarity to Ideal Solution) [8]. TOPSIS was chosen due to its popularity and extensive application across various decision-making contexts [8,31,32]. Originally developed with vector normalization, TOPSIS has since been extended with alternative normalization methods to enhance ranking consistency and discriminatory power [33]. Moreover, this study develops a conceptual link between entropy, data distribution, and normalization behavior. It demonstrates that normalization is not distribution-neutral and that entropy mediates its impact on ranking stability. These contributions advance methodological transparency and offer practical guidance for selecting normalization procedures in entropy-diverse decision problems.

The paper is organized as follows. Section 2 introduces entropy as a descriptor of the criterion data distribution. Section 3 presents a detailed theoretical analysis of selected normalization techniques and their properties. Section 4 and Section 5 provides an illustrative empirical example using TOPSIS. Section 6 concludes with a discussion of findings and directions for future research.

## 2. Entropy in Multiple-Criteria Decision Analysis

### 2.1. Shannon Entropy as a Descriptor of Criterion Information

Entropy, a fundamental concept from information theory introduced by Shannon [26], quantifies uncertainty, disorder, or information content in a system. In MCDA, it characterizes the distributional structure and uncertainty inherent in criterion values across alternatives. From this perspective, entropy captures the uncertainty inherent in criterion distributions, which affects the stability of normalized rankings. Unlike classical dispersion measures such as variance or range, which focus on the spread of values around the mean and largely depend on their magnitude, entropy captures the evenness of the distribution. In other words, entropy evaluates how uniformly values are distributed across categories or alternatives, regardless of their absolute size. This allows entropy to reveal subtle differences in the structure of the data that may remain hidden when using traditional dispersion measures [34]. Such characteristics make entropy particularly useful in MCDA, as it helps to better capture the relative differences among criteria and alternatives, where decision matrices involve heterogeneous criteria in different units and diverse data distributions [8,35,36].

Let a decision problem consist of m alternatives $A_{1},A_{2},\ldots ,A_{m}$ evaluated with respect to n criteria $C_{1},C_{2},\ldots ,C_{n}$. The decision matrix is denoted as

(1) $$D=\left[x_{ij}\right],$$

where $x_{ij}$ represents the performance of alternative $A_{i}$ under criterion $C_{j}$.

To evaluate the informational structure of a given criterion, the original values are transformed into non-negative proportions:

(2) $$p_{ij}=\frac{x_{ij}}{\sum_{i}^{m}x_{ij}}(i=1,2\ldots ,m;\;j=1,2,\ldots n).$$

This formulation implicitly assumes non-negative criterion values and reflects relative rather than absolute performance differences.

The Shannon entropy of criterion $C_{j}\;$ is defined as:

(3) $$E_{j}=-\frac{1}{\ln m}\sum_{i=1}^{m}p_{ij}\ln p_{ij},\;\;j=1,2,\ldots ,n.$$

Here, the factor $-\frac{1}{\ln m}\;$ serves as a normalization factor, ensuring that the entropy value $E_{j}$ lies in the range [0,1].

High entropy values indicate a relatively uniform distribution, implying limited discriminatory power. Low entropy reflects stronger dispersion and higher informational content. When $x_{ij}=0$, the term $p_{ij}\ln p_{ij}=0$ is conventionally assumed to be zero. To avoid zero values in practical applications, Ref. [28] proposed a modified formulation that introduces a small positive constant C, ensuring strictly positive proportions.

Entropy measures the informational capacity of a criterion to differentiate alternatives rather than its importance or preference weight. This distinction is crucial in methodological studies that aim to analyze the behavior of MCDA models under varying data structures, independently of decision-maker preferences [13,25].

Several generalized entropy measures have been proposed to capture uncertainty under different assumptions, including Rényi entropy [30], Tsallis entropy [29], and fuzzy entropy [37]. These formulations differ in sensitivity to extreme values, tail behavior, and dominance effects, and have been applied in MCDA to address incomplete, noisy, or non-extensive data environments [36,38]. Despite these developments, Shannon entropy remains widely used for its strong theoretical foundation, simplicity, intuitive interpretation, and lack of tuning parameters, enhancing transparency and reproducibility. Accordingly, this study adopts it as a representative measure of criterion data dispersion.

### 2.2. Entropy-Based Weighting: Applications and Limitations

In MCDA literature, entropy is most commonly employed as an objective weighting technique, referred to as the entropy weight method (EWM). The underlying assumption is that criteria exhibiting greater variability (lower entropy) contain more decision-relevant information and should therefore receive higher weights, whereas criteria with more uniform values (higher entropy) contribute less information and are assigned lower weights. This approach reduces the influence of subjective judgment [29,36,39,40].

Two computational schemes are commonly used for EWM: direct entropy estimation from the original criterion values and entropy estimation following normalization, typically using max–min scaling. The choice between these approaches affects the resulting entropy values and criterion weights [13,41].

The EWM has been widely applied across a broad range of domains, including management-oriented analyses [42], financial performance evaluation [43], environmental quality assessment [44], sustainable energy systems [45,46], water resources management [47], facility and location selection problems [48], urban air quality evaluation [49], and tourism performance analysis [46,50]. In these studies, EWM is frequently integrated with established multi-criteria methods such as TOPSIS, VIKOR (VIekriterijumsko KOmpromisno Rangiranje), AHP (Analytic Hierarchy Process), COPRAS (Complex Proportional Assessment), and others, to support objective criterion weighting and ranking.

The entropy method, commonly applied for objective weight determination [51], exhibits several limitations. It assumes that greater variability implies higher criterion importance, which may not reflect decision-makers’ priorities, as criteria with low dispersion can still be essential in practice. As a result, entropy-based weights may inadequately represent strategic or policy-related relevance. In addition, entropy weights are sensitive to data preprocessing, particularly normalization and scaling procedures, which can substantially affect the resulting weight distribution. This sensitivity raises concerns regarding the robustness and reproducibility of results [13]. Moreover, the method assumes independence among criteria and does not account for potential interrelationships, which may distort importance assessment in complex decision contexts. Finally, the purely data-driven nature of entropy excludes expert judgment, suggesting that entropy-based weighting is more effective when combined with subjective or hybrid approaches rather than used as a standalone method [52,53,54,55].

These limitations motivate a broader interpretation of entropy beyond its traditional role in weighting and justify its use as a descriptive analytical tool in methodological MCDA research.

### 2.3. Entropy as a Descriptive Characteristic of Criterion Data Structure and Its Methodological Role in Normalization

Beyond weighting, entropy can be interpreted as a descriptive characteristic of the decision matrix, capturing the internal distribution of criterion values. From this perspective, entropy does not prescribe importance but provides a systematic explanation of:

- the inherent discriminatory power of the criteria before aggregation,
- similarities and differences in data structure across decision problems,
- the sensitivity of MCDA results to data characteristics rather than preferences.

This interpretation is particularly relevant in methodological studies focusing on normalization, ranking stability, and rank reversal, where the objective is to analyze how MCDA techniques respond to varying data conditions rather than to support a single decision-making case.

Normalization is a fundamental step in MCDA, transforming heterogeneous criteria into comparable, dimensionless scales. Since entropy captures the distributional structure of criterion values before normalization, it provides a natural framework for analyzing how normalization interacts with data characteristics. Different techniques respond differently to dispersion, skewness, and extreme values. Consequently, criteria with identical ranges but different entropy levels may produce substantially different normalized values and inter-alternative distances, even when the original matrix is unchanged. Importantly, entropy does not modify normalization formulas directly but influences outcomes indirectly through its interaction with the mathematical properties of the methods [11].

Entropy mediates the effects of normalization on ranking stability. By capturing the structural characteristics and inherent uncertainty of criterion distributions, entropy helps explain why alternative normalization schemes may produce different rankings, even when weights and decision-maker preferences remain unchanged. Consequently, joint consideration of entropy and normalization provides a theoretically grounded framework for evaluating ranking robustness in MCDA. This perspective directly motivates the empirical analysis that follows and underpins the comparative assessment of normalization techniques presented in the next section.

In this study, entropy is used solely as a descriptive measure of data structure, distinct from its traditional role in objective weighting in MCDM, to avoid potential confusion.

## 3. Normalization Techniques in MCDA—Overview and Conceptual Framework

### 3.1. Conceptual Foundations of Normalization in MCDA

Normalization is a fundamental preprocessing step in MCDA, transforming criterion values expressed in different units, ranges, or magnitudes into a common and comparable scale. Without proper normalization, criteria with larger numerical values may dominate aggregation results, regardless of their actual importance. Effective normalization, therefore, ensures balance, interpretability, and fairness in multi-criteria evaluation.

From a methodological perspective, normalization techniques can be broadly classified into vector, linear, and non-linear transformations [33]. Vector normalization scales criterion values with respect to the overall magnitude of observations for a given criterion, preserving relative proportions while accounting for differences in units. Linear normalization methods, such as max, max–min, and sum-based transformations, are widely used due to their simplicity, transparency, and ease of implementation. Non-linear normalization approaches, although less frequently applied, are particularly useful for data characterized by strong skewness, exponential growth, or logarithmic relationships, allowing for more flexible handling of extreme values.

In this study, seven normalization techniques commonly applied in MCDA are examined, with particular emphasis on their use within the TOPSIS framework (see Appendix A). Normalization methods have been applied in TOPSIS studies, depending on the authors and the problem context. Non-linear vector normalization has been used in two main variants: N1, applied in [11,25,56,57,58], and N2, used by [11,59]. Among linear normalization methods, the max method has been employed in variants N3 [23,25,57,59] and N4 [11,58]. The max–min method (N5) has been used in [11,25,59]. Finally, the sum method has been applied in variants N6 [23,58] and N7 [25,57,59].

Each normalization procedure explicitly distinguishes between benefit and cost criteria. A criterion is classified as a benefit criterion when higher values indicate more desirable performance, whereas a cost criterion represents situations in which lower values are preferred. For each method, the corresponding formulations for both criterion types are analyzed in the subsequent sections. This analysis will provide insights into the strengths and weaknesses of each normalization technique.

The analysis focuses on the comparative characteristics of normalization techniques, including the range of normalized values, sensitivity to extreme observations, dependence on the number of alternatives, and invariance under linear and positive affine transformations. Particular attention is devoted to the issue of rank reversal, which may arise when alternatives are added to or removed from the decision set, potentially affecting ranking stability [60,61]. As emphasized by Aires & Ferreira [62], rank reversal constitutes a central and unresolved issue in MCDM, involving diverse methods and scenarios, and continues to motivate research on the modeling and robustness of rankings.

In addition to scale and range, the internal distribution of criterion values influences normalization outcomes. Even with similar numerical ranges, criteria can differ in how uniformly their values are distributed across alternatives. This characteristic can be described using entropy, which here serves solely as a descriptor of data distribution rather than a weighting mechanism. Entropy highlights the discriminatory power of criteria and helps explain differences in normalization effects, particularly regarding ranking stability and sensitivity to rank reversal.

A normalization formula N is said to satisfy the Linear Transformation property if, for criteria $C_{i}$ and $C_{j},$ where $y_{ij}=ax_{ij}\;$ with a>0, it holds that

(4) $$N(y_{ij})=N(x_{ij}).$$

Similarly, a normalization formula *N* satisfies the Positive Affine Transformation property if, for criteria $C_{i}$ and $C_{j},$ where $y_{ij}=ax_{ij}+b$ with, a>0 and b≠0, it holds that

(5) $$N(y_{ij})=N(x_{ij}).$$

These properties describe whether normalization outcomes remain invariant under rescaling or shifting of criterion values. Overall, this analysis will provide insights into the strengths and weaknesses of each normalization technique.

### 3.2. Literature Review on Normalization Techniques and Ranking Stability in MCDA

This section reviews the main research streams on normalization techniques in MCDA, with particular emphasis on their mathematical properties, effects on ranking outcomes, and implications for ranking stability. The review provides the methodological background for the comparative analysis of normalization procedures presented in the subsequent sections.

The first direction focuses on the classification and formal properties of normalization techniques. Foundational studies distinguished vector-based, linear, and non-linear methods and analyzed their mathematical characteristics, including sensitivity to extreme values, scale dependence, and invariance under linear and affine transformations [11,19]. Jahan and Edwards [57] provided a comprehensive overview, identifying thirty-one normalization techniques and discussing their advantages and limitations in engineering decision problems. More recent surveys further systematize these methods, highlight implementation pitfalls, and propose guidelines for their appropriate use [10,18,19].

The second direction examines how normalization affects ranking outcomes. Empirical studies show that applying different normalization techniques to the same data can produce substantially different preference values and alternative rankings, even when weighting and aggregation procedures remain unchanged [20,21,22]. These differences are often due to varying sensitivity to extreme values, the number of alternatives, and the treatment of cost and benefit criteria. Rank reversal, where the relative ordering of alternatives changes due to normalization choices or modifications in the decision set, has emerged as a key concern in this research [10,60,63].

The third direction considers normalization within specific MCDA methods. Different techniques have been applied and compared across TOPSIS [8,12,13,25,64], VIKOR [65], AHP [66], COPRAS [22], SAW (Simple Additive Weighting) [11,15], ELECTRE (ÉLements pour laaide à la DÉCision-REcherche et ELimination) [11,67], PROMETHEE II (Preference Ranking Organization Method Enrichment Evaluations) [68], the Hellwig method [69], and others.

The fourth direction involves empirical evaluation and robustness analysis. Comparative studies use correlation measures, ranking consistency indices, and simulation-based experiments to assess how normalization choices influence ranking stability under different decision scenarios [15,23,24,25,70]. Max–min, vector, sum, and max normalization are among the most frequently used techniques, but are also highly sensitive to data distribution and extreme values [18]. Ranking stability is frequently assessed using ranking consistency indices, Kendall and Spearman coefficients, and mean error measures, providing quantitative support for the choice of normalization [24,56,71].

Finally, a key research stream focuses on normalization techniques in TOPSIS and offers guidance for their selection. Vector normalization is generally considered the most consistent across different problem sizes and data ranges [24,35,56,59]. However, sum normalization can serve as an effective alternative depending on data characteristics [21,28,56].

### 3.3. Normalization Procedures and Their Properties

Let $C=\left\{C_{1},\ldots {,C}_{n}\right\}\;$ denote the set of criteria and $A=\left\{A_{1},\ldots {,A}_{m}\right\}$ the set of alternatives. The performance score of alternative $A_{i}$ with respect to criterion $C_{j}$ is denoted by $x_{ij}\;$ where i=1,2,…,m and j=1,2,…,n.

Each normalization method provides distinct formulations for benefit and cost criteria and exhibits different mathematical properties, sensitivity to data distribution, and implications for ranking stability.

#### 3.3.1. Vector Normalization (N1, N2)

Vector normalization scales the performance ratings of alternatives relative to the Euclidean norm of the vector formed by all alternatives for a given criterion.

For benefit criteria, the normalized value $n_{ij}$ is calculated as:

(6) $$n_{ij}=\;\frac{x_{ij}}{\sqrt{\sum_{i=1}^{m}{\left(x_{ij}\right)}^{2}}}\;\;\;\;\;\;\;\;\;\;\;(N1,\;N2)$$

For cost criteria, two commonly used variants exist. In the first variant (N1), lower values are preferred by subtracting the normalized value from one [57]:

(7) $$n_{ij}=\;1-\;\frac{x_{ij}}{\sqrt{\sum_{i=1}^{m}{\left(x_{ij}\right)}^{2}}}\;\;\;\;\;\;(N1)$$

In the second variant (N2), reciprocal values are used [8]:

(8) $$n_{ij}=\;\frac{1/x_{ij}}{\sqrt{\sum_{i=1}^{m}{\left(1/x_{ij}\right)}^{2}}}\;\;\;\;\;\;(N2)$$

N2 cannot be applied if any $x_{ij}=0$, as this results in division by zero.

Vector normalization rescales values according to their relative contribution to the overall magnitude of the criterion vector. As a consequence, normalized values depend not only on the absolute magnitude of each alternative. They also depend on the relative distribution of values across all alternatives. The normalized values are dimensionless and lie within the interval [0,1], although the effective range depends on the ratio between extreme values.

For benefit criteria, the range of normalized values is:

(9) $$\left[\frac{\underset{i}{\min}\;x_{ij}\;}{\sqrt{\sum_{i=1}^{m}{\left(x_{ij}\right)}^{2}}};\frac{\;\underset{i}{\max}\;x_{ij}}{\sqrt{\sum_{i=1}^{m}{\left(x_{ij}\right)}^{2}}}\right]$$

For cost criteria, the ranges differ between N1 and N2. In the case of N1:

(10) $$\left[1-\frac{\;\underset{i}{\max}\;x_{ij}}{\sqrt{\sum_{i=1}^{m}{\left(x_{ij}\right)}^{2}}};1-\frac{\underset{i}{\min}\;x_{ij}\;}{\sqrt{\sum_{i=1}^{m}{\left(x_{ij}\right)}^{2}}}\right]$$

whereas for N2:

(11) $$\left[\frac{1/\underset{i}{\max}\;x_{ij}}{\sqrt{\sum_{i=1}^{m}{\left(1/x_{ij}\right)}^{2}}};\frac{1/\underset{i}{\min}\;x_{ij}}{\sqrt{\sum_{i=1}^{m}{\left(1/x_{ij}\right)}^{2}}}\right].$$

These expressions indicate that both the distribution of criterion values and the number of alternatives directly influence the normalization outcome. As the ratio $\frac{\underset{i}{\max}\;x_{ij}}{\underset{i}{\min}\;x_{ij}}$ or $\frac{\underset{i}{\min}\;x_{ij}}{\underset{i}{\max}\;x_{ij}\;}$ decreases, normalized values are compressed into a narrower portion of the [0,1] interval. Moreover, for a fixed distribution of values, increasing the number of alternatives tends to reduce the length of the effective normalized interval [11].

Vector normalization satisfies the Linear Transformation property but not the Positive Affine Transformation property [11]. Changes in the domain of criterion values, such as adding or removing alternatives, may influence normalized scores and potentially lead to rank reversal, affecting ranking stability [60].

Vector normalization is moderately sensitive to extreme values, especially in the reciprocal-based N2 variant. For criteria where most values are clustered near one end of the range (low-entropy), vector normalization compresses the differences among the remaining alternatives, making them harder to distinguish. In contrast, for criteria with a wider spread of values (high-entropy), the normalization better preserves the relative distances between alternatives. Overall, the impact on ranking stability is medium compared to other normalization methods.

Despite these limitations, vector normalization remains one of the most widely adopted approaches in MCDA due to its conceptual simplicity and compatibility with distance-based aggregation methods [11,25,56,57,58]. It should be noted that in the standard TOPSIS method [8], the normalization Equation (6) is applied to cost criteria as well. The positive and negative ideal solutions can be determined directly, without converting cost-type criteria into benefit-type criteria.

#### 3.3.2. Linear Max Normalization (N3, N4)

Linear max normalization rescales criterion values relative to the maximum observed performance.

For benefit criteria, the normalized value $n_{ij}$ is defined as:

(12) $$n_{ij}=\;\frac{x_{ij}}{\underset{i}{\max}\;x_{ij}}\;\;\;\;\;\;\;(N3,\;N4.)$$

For cost criteria, two alternative formulations are employed to reflect the preference for lower values. In the N3 variant, the normalized value is computed as [57]:

(13) $$n_{ij}=\;1-\frac{x_{ij}}{\underset{i}{\max}\;x_{ij}}\;\;\;\;\;(N3)$$

In contrast, the N4 variant uses a reciprocal relation with respect to the minimum observed value [11,58]:

(14) $$n_{ij}=\frac{\underset{i}{\min}\;x_{ij}}{x_{ij}}\;\;\;\;\;\;\;\;(N4)$$

While N3 preserves linear proportionality, N4 introduces nonlinear penalization of higher costs. These formulations cannot be applied if $\underset{i}{\max}\;x_{ij}=0,$ as division by zero would occur. Here, $\underset{i}{\max}\;x_{ij}\;$ and $\underset{i}{\min}\;x_{ij}$ denote the maximum and minimum performance ratings, respectively, among all alternatives for the j-th criterion.

A key advantage of linear max normalization is its linearity. The transformation preserves proportional differences between alternatives by scaling all values directly with respect to the maximum (or minimum in the case of N4 for cost criteria). Consequently, the relative ordering of alternatives is maintained without distortion of proportional relationships [23]. For benefit criteria, the range of normalized values obtained using linear max normalization is:

(15) $$\left[\frac{\underset{i}{\min}\;x_{ij}}{\underset{i}{\max}\;x_{ij}\;};1\right].$$

For cost criteria, the normalized ranges differ depending on the selected variant. In the case of N3, the range is:

(16) $$\left[0;1-\frac{\underset{i}{\min}\;x_{ij}}{\underset{i}{\max}\;x_{ij}}\right]$$

whereas for N4 it becomes:

(17) $$\left[\frac{\underset{i}{\min}\;x_{ij}}{\underset{i}{\max}\;x_{ij}};1\right]$$

All these ranges lie within the interval [0,1]. Importantly, their length depends solely on the ratio $\frac{\underset{i}{\min}\;x_{ij}}{\underset{i}{\max}\;x_{ij}\;}$ or $\frac{\underset{i}{\max}\;x_{ij}}{\underset{i}{\min}\;x_{ij}\;}$, between the minimum and maximum values, rather than on the internal distribution of criterion values. Unlike vector normalization, linear max normalization, the normalized range does not reflect the full internal distribution of the data. Linear max normalization satisfies the Linear Transformation property and does not satisfy the Positive Affine Transformation property [11].

Linear max normalization is highly sensitive to extreme values due to reliance on maximum or minimum scores. Low-entropy criteria amplify contrasts among alternatives with extreme values, enhancing discrimination, while high-entropy criteria yield clustered normalized scores. The overall impact on ranking stability is relatively high compared to vector normalization and shows the role of entropy in interpreting N3 and N4 [60].

The method is simple, fast, and intuitive, making it suitable when extreme values are meaningful. However, reliance on maximum and minimum values requires caution in the presence of outliers or changing alternative sets.

#### 3.3.3. Linear Max–Min Normalization (N5)

Linear max–min normalization (N5), unlike linear max normalization, max–min normalization maps all criterion values linearly onto [0,1], considering both maximum and minimum values. The worst observed performance is assigned a normalized value of 0, while the best observed performance receives a value of 1. Relative differences between alternatives are preserved proportionally within the normalized scale.

For benefit criteria, the normalized value $n_{ij}$ is defined as:

(18) $$n_{ij}=\frac{x_{ij}-\underset{i}{\min}\;x_{ij}}{\underset{i}{\max}\;x_{ij}-\underset{i}{\min}\;x_{ij}}\;\;\;\;\;\;(N5)$$

For cost criteria, where lower values are preferred, the normalization is adjusted accordingly [11]:

(19) $$n_{ij}=1-\;\frac{x_{ij}-\underset{i}{\min}\;x_{ij}}{\underset{i}{\max}\;x_{ij}-\underset{i}{\min}\;x_{ij}}\;\;\;(N5)$$

In these formulas, $\underset{i}{\max}\;x_{ij}$ and $\underset{i}{\min}\;x_{ij}$ denote the maximum and minimum performance ratings, respectively, across all alternatives for the j-criterion.

This property ensures direct comparability across criteria, regardless of their original units or magnitudes, and greatly facilitates interpretation in subsequent aggregation and ranking procedures [23]. N5 satisfies both the Linear Transformation property and the Positive Affine Transformation property [11]. This distinguishes N5 from vector, max, and sum-based normalization techniques and provides a degree of robustness with respect to simple rescaling of the original data. However, when comparing linear max normalization (N3) and max–min normalization (N5), we can observe that N3 can be regarded as a special case of N5, in which the minimum of the scale is set to a conventional absolute zero, independent of the observed data. Nevertheless, N3 and N5 are generally treated as distinct normalization methods in the literature.

Max–min normalization preserves wide dispersion for low-entropy criteria and produces clustered scores for high-entropy criteria, reflecting its interaction with data structure. While it is invariant to rescaling, the method remains sensitive to extreme values. Outliers define the maximum and minimum, which can compress other scores and affect discrimination. The fixed [0, 1] range maps values proportionally. Low-entropy criteria yield widely dispersed normalized scores, while high-entropy criteria produce tightly clustered values, reducing effective resolution. Changes in the set of alternatives may alter maxima or minima, potentially causing rank reversal. Techniques such as introducing extreme fictitious alternatives [62,72] can help stabilize rankings.

In practice, N5 is widely used in SAW and other linear aggregation methods due to its simplicity and interpretability, though careful handling of outliers and alternative set changes is required.

#### 3.3.4. Linear Sum Normalization (N6, N7)

Linear sum normalization rescales values relative to the total sum of a criterion. Larger original values dominate the normalized sum, while smaller values contribute proportionally less.

For benefit criteria, the normalized value $n_{ij}$ is computed as:

(20) $$n_{ij}=\frac{x_{ij}}{\sum_{i=1}^{m}x_{ij}}\;\;\;(N6,N7)$$

For cost criteria, two variants are typically used. In the first variant (N6), the normalized value is inverted to reflect preference for lower values.

Linear: Sum (N6) [58]:

(21) $$n_{ij}=\;1-\;\frac{x_{ij}}{\sum_{i=1}^{m}x_{ij}}\;\;\;(N6)$$

In the second variant (N7), reciprocal values are employed [57]:

(22) $$n_{ij}=\;\frac{1/x_{ij}}{\sum_{i=1}^{m}(1/x_{ij})}\;\;\;\;\;(N7)$$

N7 cannot be applied when $x_{ij}=0$ as this would result in division by zero. Consequently, careful preprocessing or alternative normalization methods are required when zero values occur in cost criteria.

Normalized values sum to one, making it suitable for assessing relative contributions. As a result, sum-based normalization is frequently employed in weighted aggregation models and proportional evaluation frameworks [58].

The range of normalized values depends on the original distribution of criterion values. For benefit criteria, the normalized values lie within:

(23) $$\left[\frac{\underset{i}{\min}\;x_{ij}\;}{\sum_{i=1}^{m}x_{ij}};\frac{\;\underset{i}{\max}\;x_{ij}}{\sum_{i=1}^{m}x_{ij}}\right]$$

For cost criteria, the ranges differ between N6 and N7.

In N6, the range is:

(24) $$\left[1-\frac{\;\underset{i}{\max}\;x_{ij}}{\sum_{i=1}^{m}x_{ij}};1-\frac{\underset{i}{\min}\;x_{ij}\;}{\sum_{i=1}^{m}x_{ij}}\right]$$

whereas in N7 it becomes:

(25) $$\left[\frac{1/\underset{i}{\max}\;x_{ij}}{\sum_{i=1}^{m}1/x_{ij}};\frac{1/\underset{i}{\min}\;x_{ij}}{\sum_{i=1}^{m}1/x_{ij}}\right]$$

Linear sum normalization (N6, N7) rescales criterion values relative to the total sum. Reciprocal transformations can be optionally applied for cost criteria. Similar to vector normalization, sum-based normalization is influenced by both the distribution of criterion values and the number of alternatives. The method is sensitive to extreme or zero values. Such values can compress other scores and reduce discrimination.

Low-entropy criteria produce asymmetric distributions that favor alternatives with extreme values. High-entropy criteria, in contrast, yield nearly uniform or clustered scores, which affects effective resolution. N6 is suitable for simple proportional inversion of cost criteria. N7 uses reciprocal scaling for stronger cost dominance. Both variants satisfy the Linear Transformation property but not the Positive Affine Transformation property [11].

The impact on ranking stability ranges from medium to high, depending on the data distribution and the set of alternatives. Both N6 and N7 normalizations are intuitive and widely used. However, care is required when handling outliers, zeros, or changes in the alternative set [58].

### 3.4. Comparison of Normalization Methods—Integrated View

All normalization methods satisfy the fundamental requirement of scale comparability by transforming heterogeneous criterion values into dimensionless measures suitable for comparison across alternatives.

Normalization mechanism. The underlying mechanisms differ across methods. Vector normalization (N1, N2) scales values relative to the Euclidean norm of the vector formed by all alternatives for a given criterion. Linear max normalization (N3, N4) scales values relative to the maximum (or minimum) observed value. Max–min normalization (N5) uses both the maximum and minimum to linearly map values into the [0,1] interval. Linear sum normalization (N6, N7) rescales values relative to the total sum of the criterion, optionally employing reciprocal transformations for cost criteria (N2, N7).

Linearity and invariance. Only max–min normalization (N5) satisfies both the Linear Transformation and Positive Affine Transformation properties, making it invariant to rescaling and shifting of criterion values. Vector normalization (N1, N2), linear max normalization (N3, N4), and linear sum normalization (N6, N7) satisfy only the Linear Transformation property; adding a constant to all values shifts normalized scores and may affect rankings.

Sensitivity to extreme values. Methods relying on maximum or minimum values (N3–N5) are highly sensitive to outliers, which may compress or distort normalized scores. Sum-based normalization (N6, N7) is also sensitive to extreme values, particularly when a small number of alternatives dominate the total sum. Vector normalization (N1, N2) is moderately less sensitive, as scaling is performed relative to the Euclidean norm. Overall, vector normalization (N1, N2) shows medium sensitivity to extreme values, max-based normalization (N3, N4) shows high sensitivity, max–min normalization (N5) moderate, and sum-based normalization (N6, N7) medium to high, depending on data distribution.

Interaction with zero values. Zero or very small values pose challenges for methods using division or reciprocal transformations. Vector normalization (N2) and sum normalization (N7) cannot be directly applied when $x_{ij}=\;0$ without prior adjustment. Max-based (N3, N4) and max–min (N5) methods handle zero values more robustly, although caution is still required when zeros coincide with extreme values. In practice, preprocessing or introducing a small positive constant may be necessary to avoid division-related issues.

Dependence on data distribution and entropy. Vector and sum-based normalizations (N1, N2, N6, N7) are strongly influenced by the internal distribution of criterion values. Low-entropy criteria dominated by a few alternatives tend to produce compressed or asymmetric normalized values, reducing discriminatory power, whereas high-entropy criteria yield more uniform scores. Max-based methods (N3, N4) preserve contrasts among dominant alternatives in low-entropy criteria. Max–min normalization (N5) maintains wide dispersion for low-entropy criteria and clustered values for high-entropy criteria, indicating that entropy governs effective resolution rather than the formal [0,1] scaling.

Sensitivity to the number of alternatives and rank reversal. All normalization methods may be affected by changes in the set of alternatives. Vector (N1, N2) and sum-based (N6, N7) methods respond to both the number of alternatives and the data distribution. In max-based and max–min normalization methods (N3–N5), the most common source of rank reversal is the change in the maximum or minimum value. This contrasts with sum-based normalization (N6–N7), where adding a new alternative alters the total sum and may cause rank reversal even without modifying the extrema [62].

Cost-type criteria treatment. Two main approaches are commonly applied for cost criteria. The first approach, linear cost inversion, rescales the original values by subtracting them from one. This preserves proportional differences and ensures that lower costs receive higher normalized values (N1, N3, N5, N6). The second approach, reciprocal cost transformation, rescales values using the reciprocal function. It increases the relative differences among higher costs while still reflecting the preference for lower values (N2, N4, N7). This distinction is relevant because linear inversion and reciprocal transformation produce different relative score distributions, especially when cost values vary considerably. As a result, the relative ordering and separation of alternatives may differ across MCDM methods employing these normalizations and may lead to different ranking outcomes.

Practical implications. Vector normalization is simple and compatible with distance-based methods, though it requires caution when handling zero or very small values (N2). Max-based normalization (N3, N4) is computationally efficient and intuitive but highly sensitive to outliers. Max–min normalization (N5) ensures uniform scaling and invariance to rescaling, although extreme values may still distort rankings. Sum-based normalization (N6, N7) emphasizes relative contributions but is sensitive to extreme or zero values, potentially affecting ranking stability.

Overall, the choice of a normalization method should consider its linearity properties, sensitivity to extreme and zero values, interaction with data entropy, and potential for rank instability. Normalization should therefore be regarded as a structural modeling decision rather than a purely technical preprocessing step. These conceptual differences form the basis for the empirical comparison of normalization-induced ranking stability presented in the next section. Selecting an appropriate normalization method involves balancing mathematical properties, data characteristics, expected ranking stability, and the practical interpretation of the transformed scale by decision-makers.

## 4. Data and Experimental Design

### 4.1. Research Framework and Objectives

The empirical part of this study systematically examines the impact of normalization methods and entropy structure on the stability of rankings in MCDA. This analysis builds on the theoretical discussion in the previous section. It focuses on how different normalization methods transform the same decision matrix and how these transformations interact with criterion distributions to influence rankings.

The primary objective of the empirical investigation is to assess ranking robustness under different normalization schemes. In particular, the study identifies conditions under which rankings remain stable and conditions that lead to ranking instability when normalization methods vary. Special attention is given to the role of entropy as a descriptive measure of criterion heterogeneity, hypothesized to moderate the sensitivity of normalized values to scale transformations and extreme observations.

Rather than treating normalization as a neutral preprocessing step, the empirical framework explicitly models it as a methodological factor that can alter inter-alternative distances and, consequently, decision outcomes. This perspective allows a more nuanced evaluation of MCDA results and contributes to the broader discussion on methodological robustness and transparency in multi-criteria decision support.

### 4.2. Decision Matrix and Data Structure

The empirical analysis is based on a decision matrix comprising ten alternatives evaluated against four criteria. Criteria C1 and C2 are benefit-type criteria, where higher values indicate better performance, whereas C3 and C4 are cost-type criteria, for which lower values are preferred. This framework reflects typical decision-making problems that consider both gains and losses simultaneously. The dataset was deliberately constructed to include both high-entropy and low-entropy criteria in order to examine normalization-induced effects under controlled conditions, rather than to model a specific real-world decision problem.

Table 1 presents the original decision matrix together with basic descriptive statistics and Shannon entropy values for each criterion.

The dataset is characterized by substantial heterogeneity in scale, dispersion, and distributional form. Shannon entropy values are computed for each criterion (Table 1). In this study, entropy is not employed as a weighting mechanism but exclusively as a descriptive indicator of the data structure, capturing the degree of heterogeneity and dominance within each criterion. High-entropy criteria distribute information evenly across alternatives, whereas low-entropy criteria concentrate information in a few observations, which amplifies sensitivity to normalization methods.

Criteria C1 and C3 exhibit relatively high entropy values, indicating a fairly uniform distribution of performance values across alternatives. Although these criteria display moderate variability, no single alternative consistently achieves the highest values across all alternatives. As a result, their informational content is evenly spread, and differences among alternatives are gradual rather than abrupt.

In contrast, criteria C2 and C4 are characterized by low entropy values combined with high variability and pronounced extreme observations. In both cases, a small number of alternatives concentrate a large share of the total informational content. For C2, exceptionally high benefit values strongly differentiate a limited subset of alternatives, while for C4, extreme cost values introduce strong asymmetry into the distribution. Such low-entropy structures are expected to amplify the effects of normalization, particularly for methods that depend on extreme values or proportional scaling.

Importantly, the original decision matrix remains unchanged throughout the analysis. All observed differences in normalized values, TOPSIS scores, and rankings arise exclusively from the application of alternative normalization procedures and their interaction with the entropy structure of the criteria.

### 4.3. Experimental Design and Procedure

The experimental design follows a controlled, stepwise procedure. First, the original decision matrix is normalized using seven normalization techniques (N1–N7), as defined in the methodological section. Benefit and cost criteria are treated according to the formulations specific to each normalization method.

Next, the TOPSIS method (see Appendix A) is applied to each normalized decision matrix using identical criterion weights (equal weights) and aggregation rules. By keeping all other modeling components constant, the experimental setup isolates the effects of normalization and entropy on ranking outcomes. Finally, ranking stability is evaluated by comparing the positions of alternatives across different normalization methods, with particular attention to instability and consistent ranking patterns.

In addition to the baseline experiment, a controlled sensitivity scenario is introduced to examine how small changes in low-entropy criteria influence ranking stability across normalization methods. Specifically, the value of the benefit-type criterion C2 for alternative A5 is reduced from 80 to 40, increasing its entropy from 0.8824 to 0.9466. This modification is intentionally limited and targets an alternative previously identified as sensitive due to extreme performance.

## 5. Results and Discussion

### 5.1. Effects of Normalization on TOPSIS Scores and Rankings

This section analyzes ranking variability induced solely by normalization choice, with all other modeling components held constant. Ranking stability is interpreted not as a normative criterion of correctness but as an indicator of methodological robustness under controlled variation. Table 2 reports the TOPSIS coefficients TN1–TN7 and Table 3 the corresponding rankings obtained under normalization methods N1–N7.

To evaluate the consistency and correlation of results obtained from TN1–TN7 based on normalization techniques N1–N7, both Pearson and Spearman correlation coefficients were calculated. The results are presented in Table 4.

Despite identical data, equal weights, and aggregation procedures, noticeable differences emerge in TOPSIS scores and final rankings. This confirms that normalization choice alone can materially affect decision outcomes.

At an aggregate level, alternatives A9 and A10 consistently occupy the top positions across all normalization methods. Their strong and well-balanced performance across both benefit and cost criteria results in high closeness coefficients regardless of the normalization scheme. This convergence indicates that alternatives with robust multi-criteria profiles are less sensitive to normalization-induced distortions.

In contrast, alternatives with mixed or asymmetric performance profiles exhibit substantial ranking variability. The most pronounced example is alternative A5, which displays the widest range of ranking positions across normalization methods. A5 achieves an exceptionally high value on the low-entropy benefit criterion C2, but simultaneously performs poorly on the low-entropy cost criterion C4. Consequently, its final ranking strongly depends on how normalization methods amplify benefit dominance and penalize extreme costs. Methods that preserve or emphasize extreme benefit values generally result in higher rankings for A5, while methods that apply stronger penalties to high cost values, particularly those using reciprocal transformations, tend to lower its ranking. This illustrates how low-entropy criteria drive ranking instability and magnify the methodological role of normalization.

Beyond these extremes, the remaining alternatives exhibit intermediate and more nuanced ranking behavior. Alternatives A2 and A8 generally occupy upper-middle positions across normalization methods, reflecting relatively strong performance combined with moderate sensitivity to normalization choice. Alternative A3 shows moderate variability, typically remaining in the middle of the ranking. Alternatives A6 and A7 consistently appear in the lower-middle part of the rankings. Although their positions shift slightly depending on the normalization method, these shifts are relatively minor compared to those observed for A5. Finally, alternatives A1 and A4 remain at the bottom across all normalization methods, confirming that normalization effects are least influential for the lowest-scoring alternatives.

**Figure A1:**
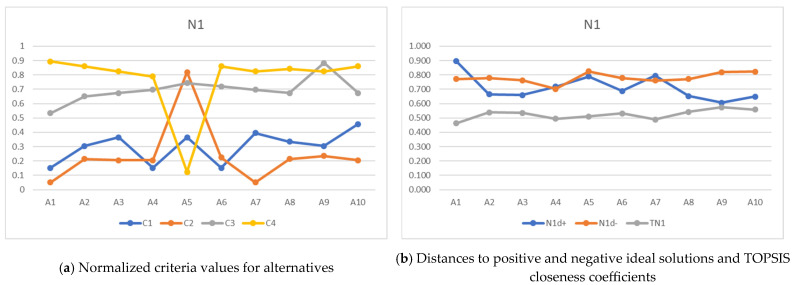
Normalization N1.

**Figure A2:**
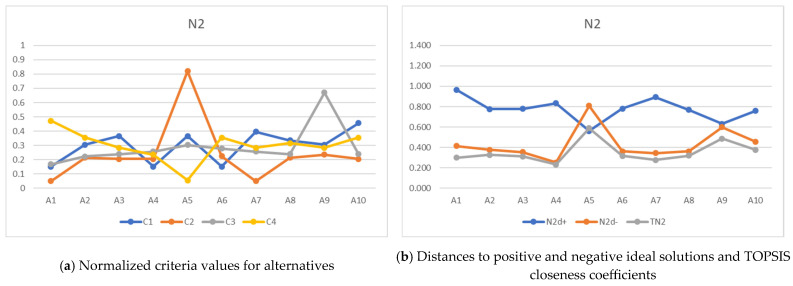
Normalization N2.

**Figure A3:**
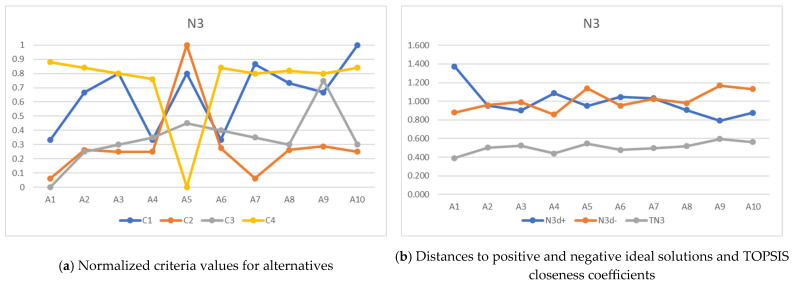
Normalization N3.

**Figure A4:**
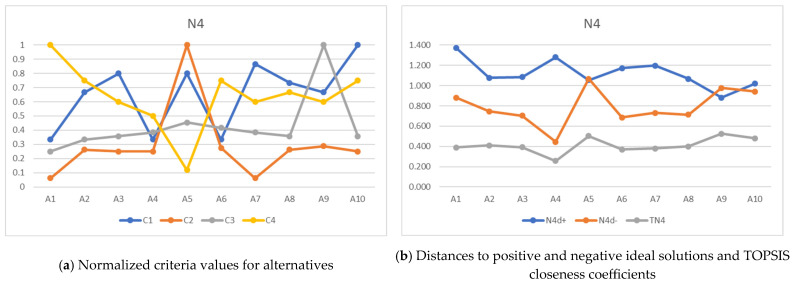
Normalization N4.

**Figure A5:**
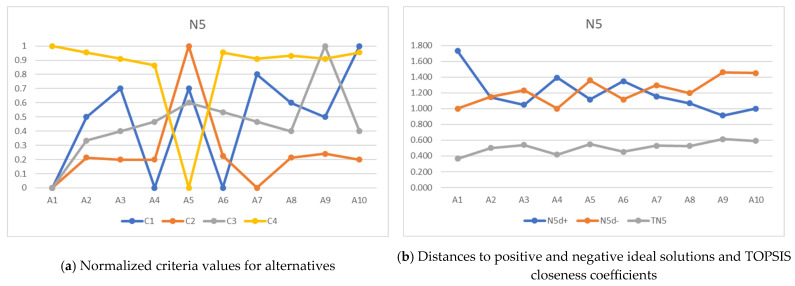
Normalization N5.

**Figure A6:**
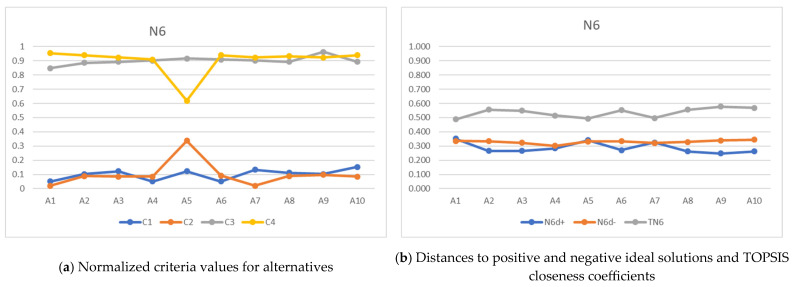
Normalization N6.

**Figure A7:**
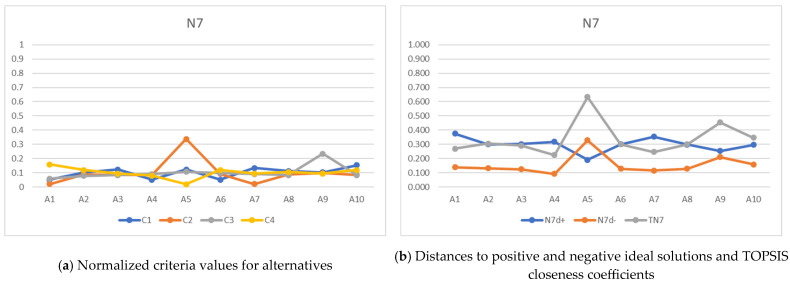
Normalization N7.

Figure A1, Figure A2, Figure A3, Figure A4, Figure A5, Figure A6 and Figure A7 (Appendix B) present the normalized criteria values N1–N7, the distances to the positive and negative ideal solutions, and the TN1–TN7 closeness coefficients. Normalized values differ according to the selected method. Differences are particularly noticeable between methods using linear cost inversion and those employing reciprocal cost transformations. The spread of TN1–TN7 values varies substantially, with the smallest spread observed for TN1 and TN6 (0.112 and 0.089, respectively) and the largest for TN2 and TN7 (0.358 and 0.407, respectively).

Reducing A5’s value in criterion C2 from 80 to 40 causes noticeable ranking shifts across normalization methods, highlighting TOPSIS’s sensitivity to data structure and normalization choice (see Table 5). A comparison of the two experimental settings shows that ranking changes are uneven across alternatives. A5 consistently drops in all normalization methods, losing between one and four positions, while other alternatives shift only slightly, typically by one position at most. This asymmetric response shows that alternatives performing very well on benefit criteria but poorly on cost criteria are the most sensitive to normalization. The pronounced drop of A5 confirms that alternatives near the decision frontier are particularly affected by small data changes, while balanced or lower-performing alternatives remain largely stable. This asymmetric response indicates that sensitivity is concentrated on alternatives that previously combined strong benefit dominance with poor cost performance.

### 5.2. Ranking Convergence, Divergence, and Ranking Stability

Analysis of ranking agreement reveals a structured pattern of convergence and divergence among the considered normalization methods. The strongest convergence occurs for rankings TN2 and TN7, which produce identical rank orders (Spearman = 1.000). High agreement is also observed between TN3 and TN5 (Spearman = 0.964), indicating that these normalization approaches lead to very similar evaluations of alternatives despite differences in their mathematical formulations.

More broadly, TN3 shows the highest overall consistency with the remaining methods, exhibiting strong correlations across a wide range of comparisons (Spearman = 0.612–0.964). This suggests that TN3 provides a balanced transformation of criterion values, preserving relative differences between alternatives while avoiding excessive amplification of extreme values, which contributes to stable ranking behavior.

In contrast, TN6 exhibits the weakest overall agreement with the other normalization methods. Its rankings show particularly low correlations with TN2, TN4, TN5, and TN7 (Spearman = 0.491, 0.479, 0.503, and 0.491, respectively). This divergence can be attributed to the combination of linear sum normalization and linear cost inversion, which respond differently to dominant observations and low-entropy criteria.

These patterns indicate that normalization differences are not merely technical. They reflect how each method processes extreme values, total sums, and cost-type criteria. Methods that handle costs nonlinearly or rely on proportional scaling tend to respond more strongly to low-entropy criteria, increasing ranking variability.

Given this structured pattern of convergence and divergence, a natural next step is to move beyond pairwise correlation analysis and provide formal support for selecting normalization methods that yield robust and representative rankings. For this purpose, a procedure for supporting multi-criteria method selection is applied (see Appendix A.2). This procedure is based on average similarity scores computed across rankings obtained with different normalization techniques. The resulting similarity measures are as follows: TN1—0.700, TN2—0.733, TN3—0.733, TN4—0.727, TN5—0.667, TN6—0.640, and TN7—0.733. These values indicate that TN2, TN3, and TN7 show the greatest agreement with the remaining methods. Notably, TN3 also achieves the highest average Spearman rank correlation (0.808) between the TOPSIS-based ranking and the other approaches. In the modified example, the similarity values increase to TN1—0.733, TN2—0.746, TN3—0.773, TN4—0.767, TN5—0.733, TN6—0.740, and TN7—0.747. Here, TN3 clearly stands out, demonstrating the strongest overall consistency. This result is further confirmed by the highest average Spearman rank correlation (0.882) with the remaining methods.

### 5.3. Role of Entropy in Explaining Normalization Effects

Entropy provides a unifying explanatory framework for interpreting the observed results. High-entropy criteria (C1 and C3) contribute to ranking stability, as their uniform distributions limit the impact of normalization-induced rescaling. Differences among alternatives remain relatively proportional across methods. In contrast, low-entropy criteria (C2 and C4) amplify normalization effects by concentrating informational content in a small number of observations. When such criteria are normalized, especially using methods sensitive to extreme values or total sums, small methodological differences translate into large changes in normalized values, distances to ideal solutions, and final rankings.

Correlation analysis confirms the role of entropy in shaping normalization outcomes. High-entropy criteria show strong alignment across normalization methods. This is reflected in high Spearman and Pearson correlations for alternatives with relatively uniform performance. In contrast, low-entropy criteria amplify the effects of methodological choices, such as the type of normalization and cost treatment. As a result, they contribute to greater ranking variability. Entropy, therefore, works together with these factors: it moderates sensitivity to extreme values, interacts with the normalization method, and, combined with cost-type treatment, affects how differences between alternatives are preserved. This confirms that ranking instability is structurally driven rather than random. Weaker correlations, such as those between TN2 and TN6, illustrate how low-entropy criteria, when combined with specific normalization and cost-handling approaches, increase sensitivity for certain alternatives.

The sensitivity experiment further confirms this mechanism: reducing the extreme value of criterion C2 for alternative A5 weakens its dominance under low-entropy conditions and leads to a consistent rank decline across all normalization methods, with the magnitude of this decline reflecting the specific sensitivity of each normalization technique.

### 5.4. Treatment of Cost Criteria and Directional Effects

The handling of cost-type criteria plays a critical role in shaping TOPSIS outcomes. Linear cost inversion, applied in TN1, TN3, TN5, and TN6, rescales cost values proportionally, ensuring consistent penalties for high-cost alternatives across normalization methods. This proportional treatment contributes to strong alignment of closeness coefficients and rankings, as reflected in high Spearman correlations between TN3 and TN5 (0.964) and between TN1 and TN6 (0.939). Whether normalization is max-based or max–min, the relative differences in costs among alternatives are preserved, leading to stable rankings even for alternatives influenced by low-entropy criteria (see also Figure A1, Figure A2, Figure A3, Figure A4, Figure A5, Figure A6 and Figure A7 in Appendix B). The reciprocal cost transformation used in TN2, TN4, and TN7 imposes non-linear penalties on high-cost alternatives. TN7 aligns perfectly with TN2 (Spearman = 1.000), while correlations with TN4 are slightly lower but still high (Spearman = 0.915), indicating generally consistent effects with minor differences in ranking outcomes.

This dual effect of nonlinear cost handling and differing normalization method increases sensitivity to low-entropy or extreme-value alternatives, resulting in weaker correlations and notable differences in absolute TOPSIS scores, as seen in TN2/TN6 (0.491).

These observations demonstrate that both the type of cost transformation and the normalization method jointly shape ranking stability, interacting with entropy to determine the sensitivity of alternatives to methodological choices. High correlations occur when cost differences are preserved proportionally, whereas nonlinear cost penalties or differing normalization bases amplify ranking variability and highlight the structural sensitivity of certain alternatives.

### 5.5. Summary of Empirical Findings

The empirical analysis demonstrates that normalization techniques significantly influence TOPSIS rankings, especially in the presence of low-entropy criteria and extreme observations. Equally important is the treatment of cost-type criteria, which interacts with entropy and normalization to determine ranking stability. While some alternatives exhibit strong ranking robustness, others are highly sensitive to normalization choice, leading to rank reversal.

These results support the central premise of this study: normalization is a substantive modeling decision rather than a purely technical preprocessing step. Entropy serves as a powerful diagnostic tool for anticipating normalization effects, assessing ranking stability, and evaluating the influence of cost treatment, thereby clarifying the impact of methodological decisions and improving robustness in MCDA applications.

### 5.6. Discussion

The primary objective of this study is not to identify a universally optimal normalization method. Rather, it seeks to demonstrate that normalization constitutes a substantive methodological choice whose effects depend on the distributional properties of criteria—quantified here through Shannon entropy—and on the treatment of cost-type indicators. By systematically examining how entropy structure interacts with different normalization schemes, the study provides diagnostic insights into ranking stability and methodological robustness in MCDA applications. In this framework, ranking stability is not interpreted as correctness or decision optimality, but as an indicator of methodological robustness with respect to controlled variations in normalization and data characteristics.

The results of the empirical experiment provide clear evidence that normalization techniques play a decisive methodological role in MCDA, especially when combined with aggregation procedures such as TOPSIS. Although normalization is often treated as a routine preprocessing step, it fundamentally shapes inter-alternative distances, affects extreme observations, and ultimately determines ranking stability.

A key insight is the explanatory power of entropy as a descriptor of the criteria data structure. High-entropy criteria exhibit relatively uniform value distributions. This limits the impact of normalization-induced rescaling and preserves proportional differences across alternatives. This effect is evident for C1 and C3, whose high entropy corresponds to consistent contributions to TOPSIS scores under all normalization schemes.

Low-entropy criteria, dominated by extreme values and strong asymmetries (C2 and C4), amplify the sensitivity of normalized values to the chosen normalization method. Methods relying on extreme values, total sums, or reciprocal transformations respond differently to these dominant observations. As a result, substantial variation occurs in TOPSIS scores and rankings.

The observed TN1–TN7 rankings confirm that ranking instability is a systematic consequence of interactions among data structure, normalization approach, and cost criterion treatment. Alternatives with balanced performance, such as A9 and A10, remain robust across methods. In contrast, alternatives with extreme benefit and extreme cost values, like A5, exhibit pronounced ranking variability. This observation is practically relevant because, in many real-world MCDA applications, the analyst begins with a performance table—crisp, interval, or fuzzy—and normalization constitutes the first modeling operation that propagates data structure into ranking outcomes.

The treatment of cost-type criteria is a critical methodological issue. Linear inversion, reciprocal transformation, and sum-based normalization impose different penalty structures on high-cost values. Differences may be negligible for high-entropy criteria but become decisive under low-entropy conditions. This reinforces that cost criteria cannot be treated as mere inversions of benefit criteria.

Overall, entropy, normalization approach, and cost-type treatment jointly mediate ranking stability. This finding underscores the need for transparent diagnostic analysis in MCDA applications and cautions against treating normalization as a neutral preprocessing step.

Building on the theoretical analysis and empirical findings, this study proposes an entropy-informed diagnostic procedure to support the selection of normalization techniques in MCDA. Although the empirical investigation was conducted using TOPSIS, the framework is not method-specific. It can be applied to other MCDA approaches that rely on normalized decision matrices and ranking-based outputs.

Proposed diagnostic procedure for selecting normalization methods in MCDA:

Step 1. Entropy assessment. Compute Shannon entropy for all criteria. Identify low-entropy criteria that may dominate the ranking. This step helps anticipate potential sensitivity to normalization and highlights the criteria for which normalization choice is most consequential (see Section 2).

Step 2. Normalization verification. Apply several normalization techniques representing different mathematical principles (e.g., linear, reciprocal, sum-based). Evaluate each method with respect to desirable theoretical properties, such as sensitivity to extreme values, preservation of proportional differences, cost-type treatment, and robustness to changes in the alternative set (see Section 3).

Step 3. Selection of candidate methods. Based on the verification, select a subset of normalization techniques that best balance robustness, discrimination, and interpretability given the structure of the decision problem.

Step 4. Comparative ranking analysis. Apply the selected normalization methods to the decision matrix. Generate rankings using the chosen MCDA method (e.g., TOPSIS or other ranking-based techniques). Compare rankings to identify alternatives exhibiting high variability. Detect systematic sensitivity patterns, particularly in the presence of low-entropy criteria and extreme performance values (see Section 4).

Step 5. Optional formal method selection. For a more formal assessment, apply the procedure described in Appendix A.2. This approach uses inter-ranking similarity measures to compare rankings obtained from multiple MCDA methods. It identifies those producing results most consistent with the others. Additionally, alternative selection criteria may be considered, such as the average Spearman rank correlation or other rank-based agreement measures.

After Step 5, the procedure does not prescribe a single mandatory decision rule. Depending on the decision context, the analyst may either:

(i) select one normalization method as representative,

(ii) adopt a consensus ranking based on several highly consistent methods, or

(iii) report a stability profile to emphasize the uncertainty of rankings.

The choice depends on whether the priority is methodological parsimony, robustness, or transparency for stakeholders.

**Figure 1:**
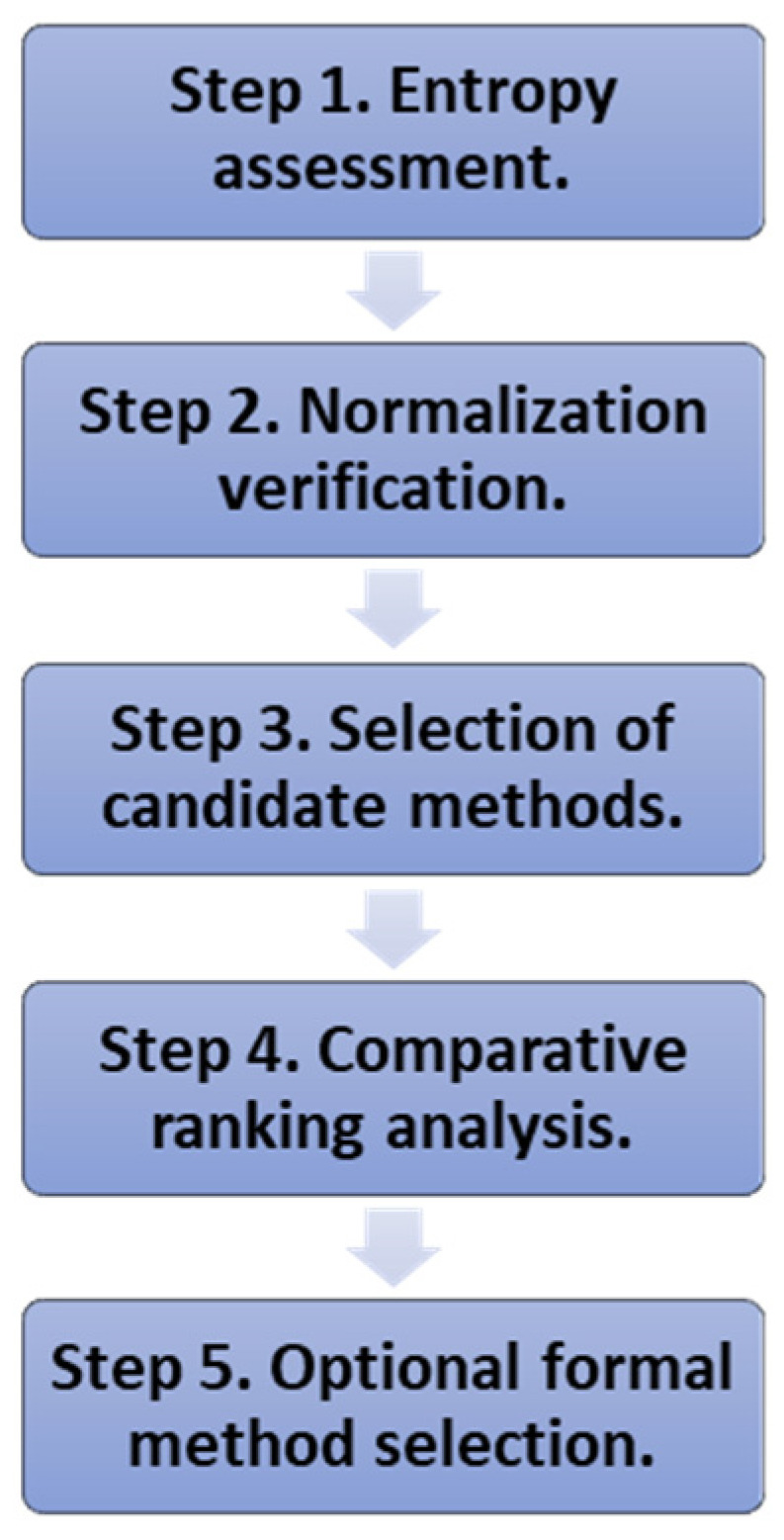
Proposed step-wise diagnostic procedure for selecting normalization methods in MCDA.

This diagnostic framework provides a practical tool for evaluating normalization effects, verifying methodological properties, and selecting normalization techniques aligned with the informational structure of the decision problem. Explicitly incorporating entropy and cost-type treatment, it supports more transparent, robust, and interpretable MCDA outcomes. The procedure is presented as a step-wise flowchart in Figure 1.

## 6. Conclusions

This study examined the role of normalization techniques in MCDA, focusing on the interplay between criterion entropy, cost-type treatment, and ranking stability. A central contribution is the use of Shannon entropy as a descriptive indicator of data structure rather than a weighting mechanism. Normalization is shown to be a substantive methodological choice that influences preference values, relative distances, and final rankings. High-entropy criteria support stable rankings, while low-entropy criteria increase sensitivity to normalization. Alternatives with extreme performances are particularly affected, as demonstrated in the TOPSIS experiment and controlled sensitivity scenario. Cost-type treatment, linear inversion, reciprocal, or sum-based normalization, further shapes rankings, especially under low-entropy conditions. The findings highlight the need to consider entropy, normalization method, and cost-type treatment jointly for transparent and robust MCDA results. The practical, entropy-based procedure for selecting normalization methods is also proposed.

This study also emphasizes the practical implications for decision-makers: by assessing entropy and understanding the sensitivity of rankings to normalization, analysts can better anticipate which alternatives are likely to experience rank reversals. This approach supports more informed and defensible decision-making, particularly in contexts where extreme values or asymmetric data are present.

Despite these contributions, the study has several limitations that suggest directions for future research. First, the analysis used a single dataset and focused on TOPSIS; extending the framework to other MCDA methods, such as PROMETHEE, VIKOR, ELECTRE, or COPRAS, would clarify its generality. Second, criterion weights were held constant; exploring interactions between entropy-based weighting and normalization represents a natural extension. Third, only Shannon entropy was considered, which captures statistical dispersion but may not fully represent uncertainty in highly skewed or heavy-tailed datasets. Alternative measures, such as Rényi or Tsallis entropy, may provide complementary insights. Fourth, the methodology assumes non-negative data, which can restrict applicability in contexts with negative or mixed-scale criteria. Finally, although synthetic data support controlled experimentation, they may not capture all complexities of real decision problems, and thus generalization should be approached cautiously.

Future research should address these limitations through systematic robustness analyses across diverse decision matrices, varying the number of alternatives and criteria, distributional characteristics, and the presence of extreme or missing values. Large-scale simulations and empirical studies would help validate the observed effects and refine practical guidance for normalization selection.

It is also important to note that the current findings are based on a synthetic dataset, which allows controlled experimentation but may not capture all complexities of real-world decision problems. Future work using diverse empirical datasets would help validate the generalizability of the observed effects.

Additionally, the proposed entropy-informed diagnostic procedure could be integrated into decision support software or applied as a preliminary step in organizational decision-making processes, providing a systematic way to anticipate and mitigate potential ranking instabilities.

## Figures and Tables

**Table 1:** Decision matrix with basic descriptive statistics and Shannon entropy values.

Alternative	C1	C2	C3	C4
A1	10	5	40	6
A2	20	21	30	8
A3	24	20	28	10
A4	10	20	26	12
A5	24	80	22	50
A6	10	22	24	8
A7	26	5	26	10
A8	22	21	28	9
A9	20	23	10	10
A10	30	20	28	8
mean	19.60	23.70	26.20	13.10
variability	34.99	83.63	26.98	94.63
min	10.00	5.00	10.00	6.00
max	30.00	80.00	40.00	50.00
max/min ratio	3.00	16.00	4.00	8.33
entropy	0.9712	0.8824	0.9824	0.8743

**Table 2:** TOPSIS coefficients obtained under normalization methods N1–N7.

Alternative	TN1	TN2	TN3	TN4	TN5	TN6	TN7
A1	0.462	0.301	0.391	0.391	0.366	0.489	0.270
A2	0.539	0.327	0.502	0.409	0.500	0.557	0.305
A3	0.536	0.313	0.524	0.393	0.540	0.549	0.291
A4	0.495	0.233	0.441	0.258	0.418	0.516	0.226
A5	0.511	0.590	0.545	0.503	0.549	0.493	0.632
A6	0.531	0.317	0.477	0.369	0.453	0.552	0.300
A7	0.489	0.278	0.498	0.379	0.529	0.497	0.246
A8	0.542	0.321	0.520	0.401	0.528	0.556	0.300
A9	0.574	0.487	0.596	0.525	0.615	0.578	0.454
A10	0.559	0.377	0.563	0.480	0.592	0.568	0.347

**Table 3:** Rankings obtained by TOPSIS under normalization methods N1–N7.

Alternative	Range TN1	Range TN2	Range TN3	Range TN4	Range TN5	Range TN6	Range TN7
A1	10	8	10	7	10	10	8
A2	4	4	6	4	7	3	4
A3	5	7	4	6	4	6	7
A4	8	10	9	10	9	7	10
A5	7	1	3	2	3	9	1
A6	6	6	8	9	8	5	6
A7	9	9	7	8	5	8	9
A8	3	5	5	5	6	4	5
A9	1	2	1	1	1	1	2
A10	2	3	2	3	2	2	3

**Table 4:** Pearson and Spearman Correlation Coefficients between TN1–TN7 under normalization methods N1–N7.

Pearson Coefficients	TN1	TN2	TN3	TN4	TN5	TN6	TN7	Spearman Coefficient	TN1	TN2	TN3	TN4	TN5	TN6	TN7
TN1	1.000							TN1	1.000						
TN2	0.368	1.000						TN2	0.673	1.000					
TN3	0.844	0.651	1.000					TN3	0.794	0.806	1.000				
TN4	0.553	0.857	0.756	1.000				TN4	0.673	0.915	0.867	1.000			
TN5	0.785	0.581	0.984	0.737	1.000			TN5	0.661	0.697	0.964	0.782	1.000		
TN6	0.935	0.056	0.614	0.314	0.559	1.000		TN6	0.939	0.491	0.612	0.479	0.503	1.000	
TN7	0.294	0.989	0.590	0.779	0.514	−0.026	1	TN7	0.673	1.000	0.806	0.915	0.697	0.491	1.000

**Table 5:** Rankings obtained under normalization methods N1–N7 after modification of criterion C2 for alternative A5.

Alternative	Range TN1	Range TN2	Range TN3	Range TN4	Range TN5	Range TN6	Range TN7
A1	9	8	10	8	10	9	8
A2	5	4	5	4	5	4	4
A3	4	7	3	6	3	5	7
A4	7	10	9	10	9	7	10
A5	10	2	7	3	6	10	2
A6	6	6	6	7	8	6	6
A7	8	9	8	9	7	8	9
A8	3	5	4	5	4	3	5
A9	1	1	1	1	1	1	1
A10	2	3	2	2	2	2	3

## Data Availability

All data are available in the manuscript.

## Appendix A

### Appendix A.1. The TOPSIS Procedure

TOPSIS [8] is a multi-criteria decision-making method for ranking alternatives based on their distances from an ideal and an anti-ideal solution. TOPSIS starts by defining a finite set of alternatives $A=\left\{A_{1},\ldots ,\;A_{m}\right\}$ and criteria ${C=\{C}_{1},\ldots ,\;C_{n}\}$, which together form the decision matrix:

$$D={\left[x_{ij}\right]}_{m\times n}.$$

The criteria are divided into benefit and cost types, and weights $w_{j}$ are assigned to the criteria such that

$$\sum_{i=1}^{n}w_{i}=1.$$

The decision matrix is normalized (using one of the normalization procedures N1–N7) to obtain the normalized matrix:

$$\bar{D}={\left[{\bar{x}}_{ij}\right]}_{m\times n}$$

The decision matrix is weighted to obtain the weighted normalized matrix:

$$\tilde{D}={\left[\;{\tilde{x}}_{ij}\right]}_{m\times n},{\tilde{x}}_{ij}=w_{j}{\bar{x}}_{ij}.$$

Based on $\tilde{D}$, the positive ideal solution and the negative ideal solution are defined as

$$A^{+}=[\;\underset{i}{\max}\;{\tilde{x}}_{ij}\;for\;the\;benefit\;criteria;\;\underset{i}{\min}\;{\tilde{x}}_{ij}\;for\;the\;cost\;criteria]$$

and

$$A^{-}=[\;\underset{i}{\min}\;{\tilde{x}}_{ij}\;for\;the\;benefit\;criteria;\;\underset{i}{\max}\;{\tilde{x}}_{ij}\;for\;the\;cost\;criteria].$$

For each alternative $A_{i}$, the distances from the ideal $d_{i}^{+}$ and anti-ideal solutions $d_{i}^{-}$ are computed (using the Euclidean metric):

$$d_{i}^{+}=\sqrt{{\left({\tilde{x}}_{ij}\;-{\tilde{x}}_{j}^{+}\right)}^{2}\;\;}{\;and\;d}_{i}^{-}=\sqrt{{\left({\tilde{x}}_{ij}\;-{\tilde{x}}_{j}^{-}\right)}^{2}\;.\;}$$

Finally, the relative closeness to the ideal solution is determined as

$$T_{i}=\frac{d_{i}^{-}}{{d_{i}^{-}+d}_{i}^{+}}$$

and alternatives are ranked in descending order of $T_{i}.$

### Appendix A.2. Procedure for Supporting Multicriteria Method Selection [73]

The procedure is based on inter-ranking similarity measures. It involves comparing the rankings of a set of m alternatives, $A_{1},\;A_{2},\ldots ,\;A_{m}$, obtained by applying v different multi-criteria methods. As a result, v distinct rankings are generated. These rankings are then compared pairwise. The comparison outcomes are interpreted as inter-ranking similarities, quantified using the ranking similarity measure $m_{pq}$, defined as follows:

$$m_{pq}=1-\frac{2\sum_{i=1}^{m}\left|c_{ip}-c_{iq}\right|}{m^{2}-z},\;p,\;q=1,\;2,\;\ldots ,v,$$

where $c_{ip}$—represents the position of the i—th alternative in the ranking generated by the p—th method; $c_{iq}$—represents the position of the i—th alternative in the ranking generated by the q—th method; $z=\left\{\begin{array}{c} 0,\;m\in P\\ 1,\;m\;\notin P \end{array}\right.$ where P denotes the set of even natural numbers.

All pairwise inter-ranking similarities can be aggregated into the matrix M:

$$M=\left[m_{pq}\right]=\left[\begin{array}{c} \begin{array}{cc} \begin{array}{cc} 1 & m_{12}\\ m_{21} & 1 \end{array} & \begin{array}{ccc} m_{13} & \ldots & m_{1v}\\ m_{23} & \ldots & m_{2v} \end{array} \end{array}\\ \begin{array}{cc} \begin{array}{cc} \vdots & \vdots \\ m_{v1} & m_{v2} \end{array} & \begin{array}{ccc} \vdots & \vdots & \vdots \\ m_{v3} & \ldots & 1 \end{array} \end{array} \end{array}\right]$$

To assess the overall similarity of the ranking obtained using the p—th multi-criteria method with respect to all remaining rankings, it is sufficient to compute the average of the elements in the p—row (or column) of matrix M, excluding the diagonal element.

This value, denoted by $u_{p}$, is given by:

$$u_{p}=\frac{1}{v-1}\sum_{\begin{array}{c} q=1\\ p\neq q \end{array}}^{v}m_{pq},\;p,\;q=1,\;2,\;\ldots ,v.$$

Finally, the multicriteria method corresponding to the highest average similarity value is selected:

$${\bar{u}}_{p}=\underset{p}{max}\;u_{p}$$

This approach ensures that the chosen method produces rankings most consistent with the others.

## Appendix B

Figure A1, Figure A2, Figure A3, Figure A4, Figure A5, Figure A6 and Figure A7 present normalized criterion values for alternatives A1–A10, their distances to the positive and negative ideal solutions, and the corresponding TOPSIS closeness coefficients (TN1–TN7) under normalization methods N1–N7.
